# Study of the Influence of the Manufacturing Parameters on Tensile Properties of Thermoplastic Elastomers

**DOI:** 10.3390/polym14030576

**Published:** 2022-01-31

**Authors:** Bàrbara Adrover-Monserrat, Jordi Llumà, Ramón Jerez-Mesa, J. Antonio Travieso-Rodriguez

**Affiliations:** 1 Mechanical Engineering Department, Escola d’Enginyeria de Barcelona Est, Universitat Politècnica de Catalunya, 08019 Barcelona, Spain; barbara.adrover@upc.edu (B.A.-M.); ramon.jerez@upc.edu (R.J.-M.); 2 Materials Science and Metallurgical Engineering Department, Escola d’Enginyeria de Barcelona Est, Universitat Politècnica de Catalunya, 08019 Barcelona, Spain; jordi.lluma@upc.edu

**Keywords:** additive manufacturing, fused filament fabrication, tensile tests, thermoplastic elastomers

## Abstract

Additive manufacturing (AM) has increased its field of application, not only for prototypes but also for final parts. Therefore, the need to study new materials is currently growing. This paper aims to study the effect of the printing parameters used in two different thermoplastic elastomers (PEBA 90A and TPU 98A) subjected to tensile tests, evaluating a competent alternative to the currently most used 3D printed materials. To achieve it, a full factorial design experiment is applied to analyze the influence on the tensile responses of two printing parameters: the layer height and the fill density. In addition, an analysis of variance (ANOVA) is used to describe the relations among the parameters and the mechanical responses obtained. Moreover, assessment of damping properties was done. Results show that each thermoplastic elastomer should be studied separately, although the proposed methodology can be used for each material independently of their nature. Finally, a correlation between the printing parameters and the mechanical behavior of TPU 98A and PEBA 90A was found: the layer height and the infill are statistically influential parameters for both materials.

## 1. Introduction

Additive manufacturing (AM) is a group of versatile fabrication processes that allow users to print layer-by-layer prototypes and final parts of complex geometries. AM techniques have been progressively developed recently due to their ease and great potential to generate complex shapes and integrate the design and manufacturing phases of a part. One of the most popular AM technologies, due to its low costs and a wide range of available materials, is called material extrusion (MEX) or fused filament fabrication (FFF).

There are many studies that examined the effect of printing parameters used in MEX on different mechanical tests of samples printed with thermoplastic materials such as PLA, ABS, or composites. Laureto et al. [1] quantified the variations of ultimate tensile strength and yield strength of FFF printed components using both type I and IV geometries of the tensile ASTM D638 standard. They printed samples of poly lactic acid (PLA) and varied some printing parameters (e.g., layer height, printing speed, printing temperature, and flow). To study the anisotropic mechanical properties variances, they compared different orientations. A comparison of samples built using the same printing parameters but with different orientations showed that vertical specimens had an ultimate tensile strength 47.9% less than the horizontal ones. Fayazbakhsh et al. [2] worked also with PLA parts, printed with and without considering the impact of defects. They studied the directional properties of FFF specimens according to the ASTM D638 standard and investigated the impact of gaps on the mechanical responses obtained through tensile tests. In their investigation, it was concluded that the gaps situated transverse to the loading direction reduced the tensile strength by 20.5%, the response of the modulus by 9.6%, and the response failure strain by 11.5%. Fernandez-Vicente et al. [3] studied the influence of two controllable variables, such as pattern and density of the infill, in order to evaluate the strength of ABS pieces. They concluded that the change in infill density mainly determines the tensile strength, achieving higher tensile strength using a 100% infill, as the pattern causes a variation of less than 5% in the maximum tensile strength. Domingo-Espin et al. [4] used the Taguchi methodology to study the influence of five different printing parameters (layer height, nozzle diameter, infill density, printing speed, and infill patterns) of ABS samples subjected to fatigue tests. Their results showed that the infill density is the most important parameter, and a honeycomb pattern showed longer lifespans. Zandi et al. [5] followed the same research methodology for a composite material (PLA-wood) and considered the influence of four printing parameters (layer height, fill density, printing velocity, and orientation) through a L27 Taguchi orthogonal array. They found the best combination in terms of mechanical properties, being 75% fill density, 0° Z-axis orientation, 0.4 mm layer height, and 40 mm/s velocity. Along the same lines, Ferreira et al. [6] considered that the materials produced by 3D printing behave like laminates formed by orthotropic layers. They printed specimens of PLA reinforced with short carbon fibers and studied the effect of printing layers in different orientations (0°, 90°, ±45°), including the tensile and shear properties of the composite material. Their research concluded that the printing orientation directly affected the mechanical properties analyzed as well as the length of the chopped fibers. 

These studies focus their investigation on thermoplastic materials. However, some other researchers have recently investigated 3D printable materials with elastomeric properties following the same lines of the studies noted above. These materials combine the thermoplastic nature of polymers along with high elasticity. Hence, these types of materials, which might be similar to soft tissues because of their mechanical properties, have gained interest in biomedical research [7,8]. Bachtiar et al. [9] characterized the elastomer PCU-Sil processed through 3D printing, suggesting possible biomedical applications. To do so, they studied the rheological and thermal properties, determining the two different glass transition temperatures of the material: the temperature that leads to a significant weight loss and the one in which the viscosity of the material change. This study led them to find a set of possible printing parameters, showing the limit of temperature that could be used to manufacture their samples. After the thermal analysis, they studied the quasi-static and the cyclic mechanical behavior with printed specimens following the ASTM D638 and ASTM D7791 standards. Finally, they state that PCU-Sil achieves a Young’s modulus of 6.9 MPa and a failure strain of 4567%. Consequently, PCU-Sil shows soft mechanical properties with good deformation. Lin et al. [10] measured the effect of nozzle height on packing geometry of cross sections and surface features by SEM of an FFF-printed elastomer. They noted that the fatigue properties of printed parts decreased when the samples have generated voids. They also studied the bonding between layers, suggesting the importance of the nozzle height in order to reduce the internal voids of the printed parts. Eventually, they concluded that the presence of voids diminishes tensile properties of the material. Koo et al. [11] studied the mechanical properties of a 3D-printable TPE reinforced by cellulose nanocrystals prepared with the process of in situ polymerization. After studying the thermal properties of the composite, they submitted the samples to tensile tests and pointed out that conventional 3D printing materials that have elastomeric properties might have problems concerning the interfacial adhesion between the layers. This is because diffusion between layers can be challenging due to their fast solidification. Robinson et al. [12] enabled a full characterization of a co-polymeric material (TPE) via a conventional material testing machine. They studied the stress-strain curve of the material considering some manufacturing parameters such as nuzzle diameter, print speed, bed and extruder temperature, and layer height. They used the samples with rectilinear pattern for the tensile tests, although they also designed a honeycomb to validate their methodology. In their results, they highlight the importance of the inter-layer effect and the need of new studies of this emerging technology. A study of dependence of temperature and humidity for an ether-based polyurethane elastomer was done by Kanyanta et al. [13]. Kanyanta characterized an elastomer for biomedical applications by performing tensile tests in different ambient conditions. They concluded that it is important to mimic the conditions of the intended applications of the elastomers, as the results depended mainly on the humidity of the room. Because of the need to use TPUs in many applications because of their good mechanical properties, Kim et al. [14] obtained a TPU without plasticizers to decrease its hardness without deteriorating its tensile strength and abrasion resistance. They achieved their goal by combining a TPU with rubber blends.

There are a large number of studies that have examined the mechanical responses of PLA samples printed by AM [1,2] as well as for ABS specimens [3,4] even with new composites materials [5,6] such as PLA reinforced with wood fibers, examining the results with a statistic analysis. Moreover, most of the tests performed in these cases were tensile tests. Nevertheless, this group of thermoplastic materials have certain limitations depending on their application, such as their lack of flexibility and deformation. Today, the additive manufacturing process is emerging, and some issues need to be solved by characterizing innovative materials capable of covering gaps that other materials may not cover. Therefore, some research groups have started studying the mechanical behavior of non-commercial silicones and reinforced thermoplastic elastomers [7,8,9,10,11,12,13,14]. However, there is no complete information for commercial filaments with elastomeric properties. 

In this light, to mechanically characterize two innovative commercial thermoplastic elastomers, the current research has two main purposes. The first one is to study an optimal printing configuration concerning two design parameters: the layer height and fill density. To conduct it, specimens of TPU98A and PEBA90A, manufactured by Fillamentum Company, are printed with the technique of fused filament fabrication (FFF). The printed samples will be submitted to tensile tests to obtain the Young module’s and yield strength for each material. Eventually, the effect of the printing parameters on the response of the material will be set to an analysis of variance (ANOVA). To compare both materials in terms of field application, a damping factor of each material was evaluated. For its second purpose, this paper attempts to determine whether the results of materials with elastomeric properties are comparable to thermoplastics, by contrasting the effects of the printing parameters on the mechanical responses between the thermoplastics studied in the literature and the elastomeric thermoplastics studied in this article. Thus, a work methodology based on the ASTM D638 standard along with a design of experiments (DoE), used to optimize the number of specimens manufactured, is presented.

The main novelty of this paper is the comprehensive study of thermoplastic elastomers considering the real construction of specimens by analyzing the effective material deposition achieved through FFF, assuming that the viscoelasticity of these materials can affect the actual construction of the workpieces. Indeed, the chemical and thermomechanical properties of these kinds of materials question the suitability of previous results obtained for conventional materials such as PLA or ABS to analyze the deformation and failure mechanisms that explain their behavior under tensile stress. The results obtained in this paper are very important to the additive manufacturing industry because the mechanical behavior of two different thermoplastic elastomeric materials, which are gaining interest, is evaluated. Therefore, this research addresses the needs of the MEX users by characterizing new materials.

## 2. Materials and Methods

Two different thermoplastics with elastomeric behavior were studied in this research. A tensile test was performed to determine the mechanical properties of each material. PEBA 90A (Fillamentum Manufacturing Czech s.r.o, Hulin, Czech Republic) is based on polyamide, whereas TPU 98A (Fillamentum Manufacturing Czech s.r.o, Hulin, Czech Republic) is based on polyurethane. Thermoplastic elastomers are copolymers with soft amorphous and hard crystalline regions. Particularly, these materials result in a combination of two characteristic behaviors: high elasticity and a thermoplastic nature (such as processing temperatures above melting temperatures and recyclability).

### 2.1. Thermal Characterization

First, the thermal properties of the materials were evaluated to guide the manufacturing process and assess whether hot extrusion affects the material’s behavior. In order to see the effects of the extruding process on the material, a differential scanning calorimetry (DSC) Mettler Toledo DSC 3+ (Mettler Toledo, Greifensee, Switzerland) and a thermogravimetric analysis (TGA) TA Instruments SDT Q600 (TA Instruments, New Castle, DE, USA) were performed in two kinds of specimens on both materials: as-received and after one cycle of extrusion. 

The first important result of the thermal characterization is that there were no differences between the behavior of the material extruded and not extruded, because both analyses resulted in the same values. 

From the DSC test, the melting temperatures of TPU and PEBA were found to be 176 °C and 150 °C, respectively. In addition, from the TGA test, the degradation temperature of TPU was observed at 352 °C, and at 446 °C in case of PEBA. In this regard, the printing temperature was set at 240°C for both materials. At this temperature, specimens present a loss of mass of 0.3% for TPU and 0.6% for PEBA, which are not significant quantities.

Therefore, it can be concluded that the process of material extrusion does not affect the thermal behavior of either material regardless of their processing through hot extrusion.

### 2.2. Tensile Testing Characterization

**Figure 1:**
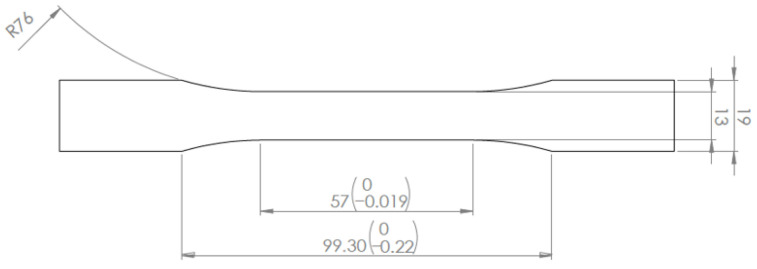
Dimensions and shape of specimens manufactured for tensile testing, 7 mm thickness.

The samples were designed with Solidworks and they were subjected to tensile tests using a universal testing machine, Zwick Allround 5 kN (ZwickRoell GmbH & Co. KG, Ulm-Einsingen, Germany), under dry-room temperature conditions. The shape and dimension of the samples are defined as per the ASTM D638 standard (Figure 1).

An STL file was exported from the design software, as the printing parameterization software can interpret this file. After the STL conversion, Simplify3D software 3.0.2 (Cincinnati, OH, USA) was used to set the printing parameters and to slice the 3D model generating a G-code. The G-code file defines the trajectory of the extruder and bed of the printer during the manufacturing process, as well as the velocity and the amount of material extruded and all the printing parameters concerning the printer. A MEX printer Ender Pro-3 (Shenzhen Creality 3D Technology Co., Ltd., Shenzhen, China) was used to extrude the material layer by layer for specimens’ development.

#### 2.2.1. Full Factorial Experimental Design

To minimize the costs and increase the productivity, this study aims to find an optimal combination of printing parameters that maximize the mechanical performance and reduce the amount of material used. To deal with the interpretation of the tests, a design of experiments (DoE) was applied. This paper studies the influence of the layer height and the fill density, as these parameters may have high impact on the mechanical properties of elastomeric materials, as according to previous research, the lower number of gaps in specimens tend to maximize its mechanical resistance [3,13,15]. 

The layer height is defined as the distance between the bed and the nozzle. A reduction in the layer height produces thinner layers and, consequently, increases the printing time. The fill density is defined as the amount of material deposited between the outer shells. A reduction of fill density leads to decrease the amount of material and a reduction of printing time.

In the present research, a full factorial design with a center point was used to evaluate two factors (layer height and fill density) and two levels per factor (low and high) plus the center point (medium) (Table 1). A total of five combinations were required in the design of experiment. In addition, five replications were used for each combination. Therefore, 25 experiments for each material were performed and evaluated.

#### 2.2.2. Preliminary Study of Inter and Intra-Layer Bonding

Because the adhesion capacity between intra-layer and inter-layer bonding is very relevant for the mechanical performance of the resulting workpieces [12,16], a preliminary study to address this issue was performed. As it was found in previous studies that there is a correlation between the existing gaps of the printed samples and their deformation/mechanical responses [12,16], the samples manufactured in this study were evaluated from this perspective. The combinations of layer heights and fill density used in this study were printed in five-layer cubic samples. Their cross sections were then observed with a Nikon Optiphot PFX microscope (Tokyo, Japan) in order to compare the necks created between filaments during the solidification process of the layers once deposited on the heated bed. The results are shown in Table 2. 

The images were then evaluated in a Matlab (Mathworks, Natick, Massachusetts, USA) routine to compute the effective area of material (% of material with respect to the square image analyzed). To define the effective area of material, a binarization process was performed manually on the images and the percentage of material was found by counting all black and white pixels. It was found that more material is deposited when printing with TPU than with PEBA at equal printing parameters. For instance, with a 0.2 mm layer height and 100% infill, the former presents a 95% of effective material area and the latter 84%. However, if the layer height increases to 0.3 mm, the effective area of material decreases for TPU. Specifically, it falls to 83%, whereas in PEBA it falls to 80%. This indicates that PEBA is less sensitive to changing the layer height when printing in terms of effective material quantity. The gaps created by the printing routine can cause failures of the samples tested due to a discontinuity of the resistant material. This result is consistent with those obtained in previous research [3], and therefore is considered relevant to understand the results obtained during the main stage of investigation.

From the images in Table 2, TPU geometrically behaves more like a conventional thermoplastic. It respects its shape when the layer height is increased. For low layer heights, the shape of the cross sections of the filaments is elliptical. When the layer height is increased, its shape becomes more circular. This effect is not emphasized in the case of PEBA. Its filaments remain in a circular shape for both settings. This observation agrees with the previous image analysis to obtain the effective area of material, as elliptical filaments result in lower porosities. This state suggests that the viscoelasticity of PEBA is higher than TPU, because when PEBA cools down, filaments show higher elastic behavior and the memory effect of layer height on the cross section of the filaments is lost.

Partial neck growths are found in both materials. TPU has longer necks for lower values of layer height but weaker necks for higher layer heights. PEBA also has longer necks for a layer height of 0.2 mm, but the bonding created for 0.3 mm is still strong.

#### 2.2.3. Experimental Setup and Data Analysis

The testing process was performed using a universal testing machine, Zwick Allround 5 kN, working with a speed of 20 mm/min. The data acquisition system used was formed by an HD camera Casio EX-F1 (Casio Computer Co. Ltd. Tokyo, Japan) at 60 Hz sampling frequency, which collects information of the displacement of the material, and a class 0.5 load cell of 5 kN that collects the tensile force at every step of the test, also at 60 Hz. A switch-controlled flash was used with two purposes: to illuminate the working area and to synchronize the data from both sources that collect information.

In addition, the initial cross section of all the samples was calculated achieving the average of three different sections using a digital palmer. The weight of all the specimens was also measured to control and reduce the possible manufacturing inaccuracy due to process problems such as under-extrusion.

The strain-stress curve of each sample was obtained from the data obtained with a data acquisition system: the values of strain were calculated from the frames recorded with the camera. The displacements tracked from the pixels that formed each frame were analyzed. The deflection was calculated and turned into real deformations. The stress values were obtained from data acquired by the testing machine. A Matlab script was used to compute and analyze the data and obtain each curve. First, a digital image correlation (DIC) measurement was performed to define the deformations of the samples. The strain-stress curve was then represented. Finally, the ASTM D638 standard was followed to calculate the Young’s modulus and yield strength from the strain-stress curve, as these two parameters characterize the tensile behavior of each specimen studied.

The results were analyzed using Minitab and subjected to an ANOVA test to evaluate the influence of the variable parameters set in the DoE.

### 2.3. Damping Evaluation

The damping of each material was evaluated as the two materials have different properties: PEBA is meant to be used in applications where vibrations should be transmitted, but TPU amortizes the input vibrations. In order to characterize the damping behavior of the MEX materials PEBA and TPU, six specimens (three of each material) were printed. All samples manufactured for the damping test had prismatic shape and their dimensions were 80 × 10 × 40 mm.

#### 2.3.1. Experimental Setup

Each specimen was secured with a clamp, with the condition being a cantilever beam. To evaluate the influence of the geometrical characteristics on the damping factor, two different free lengths, 60 mm and 70 mm, were used for each specimen. 

#### 2.3.2. Vibration Monitoring

The specimen free end was deformed and then it was released in order to produce free vibrations until the movement died away as a consequence of internal damping. This movement was monitored with a laser vibrometer system, composed of a Polytec OFV-505 head sensor and a Polytec OFV-5000 controller, (both from Polytec, Baden-Württemberg, Germany) The vibration was recorded by an LMS PIMENTO measurement system (LMS International, Leuven, Belgium). Subsequently, the time histories were exported to Matlab where the results were analyzed and processed.

## 3. Results and Discussion

Results for tensile tests and damping evaluation are described, analyzed, and discussed in this section. 

### 3.1. Tensile Testing

Table 3 contains the mechanical parameters obtained with the stress-strain curve for each configuration tested. These results are subjected to an ANOVA analysis, as explained in the next section.

#### 3.1.1. Analysis of Variance of Tensile Tests

The influence of each factor defined on the DoE is evaluated statistically by subjecting the results obtain in the tensile tests to an ANOVA. To assess the statistical influence, a *p*-value associated with each factor is calculated and compared to a significance level of 5%. Therefore, a factor will be considered to have a significant effect on the response when its associated *p*-value is lower than 0.05. Second order interactions are done concluding possible interactions between factors.

##### TPU 98A

When studying TPU 98A, both factors, layer height and infill, emerge as statistically significant for the two responses considered (Young’s modulus and yield strength). Moreover, the interaction of both factors also influence both responses.

1. Young’s Modulus

**Figure 2:**
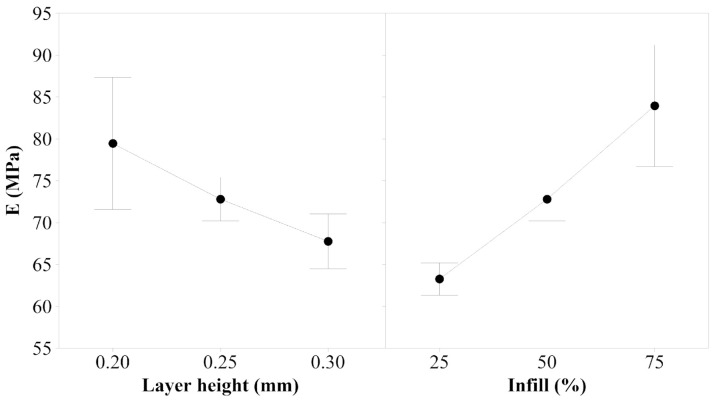
Main effects plot for Young’s modulus of TPU 98A.

**Figure 3:**
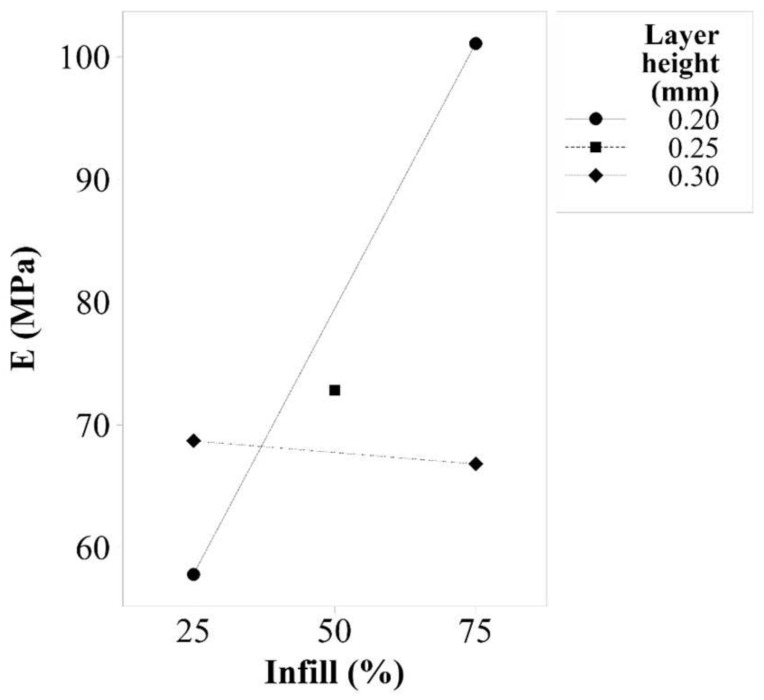
Interaction effects plot for Young’s modulus of TPU 98A.

Layer height has an associated *p*-value of 0.016, which is a statistically significant factor. Infill is also a significant factor for Young’s modulus response, as its *p*-value is 0.001. Figure 2 shows that to increase the value of Young’s modulus for TPU 98A, a layer height of 0.20 mm and an infill of 75% should be used. However, it is important to consider the interaction between these two parameters, as it has a *p*-value of 0.000. The first important thing to notice in the interaction effect plots shown in Figure 3 is that the factor of layer height of 0.20 mm is the more sensitive, as it shows a bigger slope than the other parameters. The interaction shows that the layer height of 0.30 mm provides more stiffness to the samples when a 25% infill is used, but it decreases the stiffness when higher values of infill are used.

2. Yield Strength

**Figure 4:**
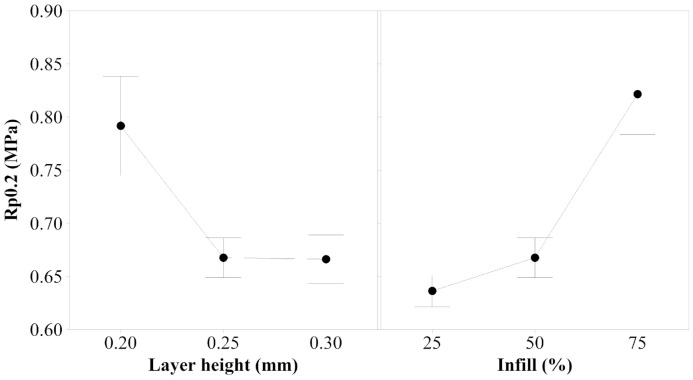
Main effects plot for yield strength of TPU 98A.

**Figure 5:**
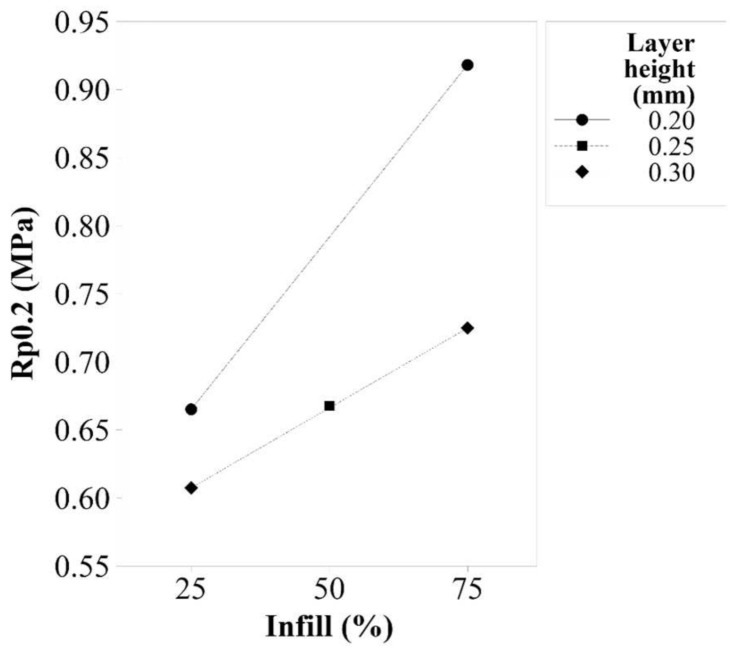
Interaction effects plot for yield strength of TPU 98A.

When the response of yield strength is analyzed, it is seen that the behavior of the factors is similar to its behavior already examined for Young’s modulus. Both factors, layer height and infill, represented in Figure 4, are significant factors that have *p*-values of 0.000. There is a statistically significant interaction between this pair of factors (*p*-value = 0.009) that shows that when the layer height adopts the value of 0.20 mm, its behavior is the most sensitive (Figure 5). An interaction is observed because a low layer height paired with a low infill produces a low value of Young’s modulus. Nevertheless, a low value of layer height combined with a high value of infill results a better value of the mechanical response than when a high value of layer height is used.

##### PEBA 90A

The results of PEBA 90A are analyzed in this section. The ANOVA shows that layer height and infill are statistical factors for Young’s modulus and yield strength. In this case, there are no significant interactions between the two factors considered.

1. Young’s Modulus

**Figure 6:**
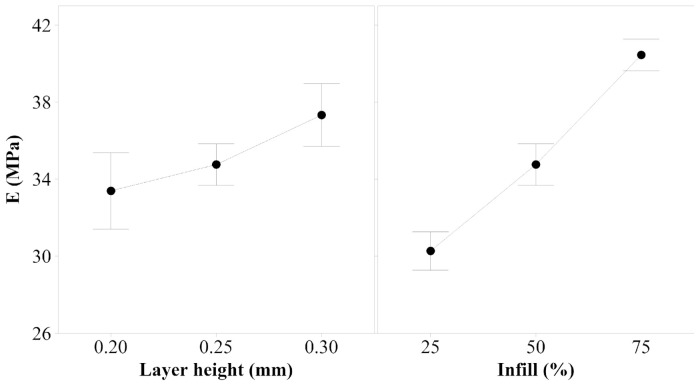
Main effects plot for Young’s modulus of PEBA 98A.

Figure 6 shows the main effects plot for Young’s modulus. The analysis of this plot, and considering the *p*-values obtained, which is 0.000 for both factors, expose the optimal parameter’s set, in terms of Young’s modulus response for tensile forces. Thus, samples should be printed with 0.30 mm layer height and 75% infill to enhance the stiffness of the samples.

2. Yield Strength

**Figure 7:**
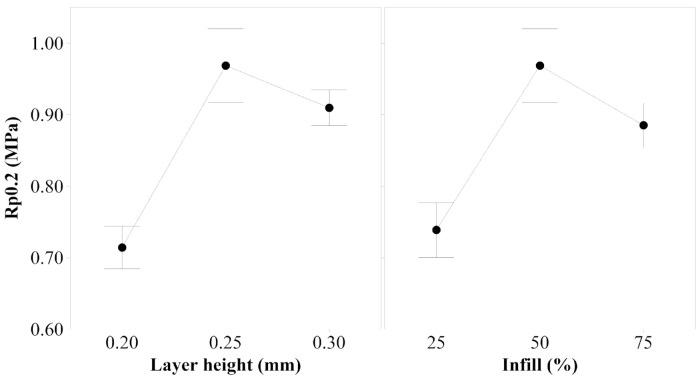
Main effects plot for yield strength of PEBA 98A.

According to the results obtained, both layer height and infill, are statistically significant for yield strength. The *p*-value is 0.000 for both factors, which indicates that the mechanical response of yield strength highly depends on the values selected for the factors when the samples are printed. Figure 7 illustrates that to observe the differences between 0.25 mm and 0.30 mm layer height, as well as 50% and 75% infill, the variance of the responses should be reduced by increasing the number of samples printed and tested. This study demonstrates that the minimum value of the range of the two factors analyzed minimizes the yield strength, but it cannot be concluded which value maximizes this property.

### 3.2. Damping Evaluation

**Figure 8:**
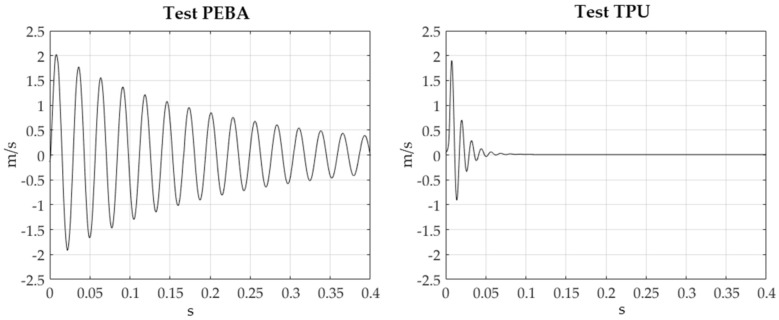
Velocity versus time corresponding to a specimen of each material.

The different measurements corresponding to the different tests were analyzed using Matlab. The monitored vibration parameter by the Polytec OFV-505 head sensor was velocity. Figure 8 details the velocity versus time corresponding to a specimen of each material (right picture corresponds to TPU and left picture corresponds to PEBA). The vibrating signal vanishes much earlier in the TPU than in the PEBA. This indicates that the TPU’s damping is much higher that PEBA’s. Therefore, PEBA will be used in applications where vibrations must be transmitted and TPU will be used in applications that require damping or vibrations are amortized. 

In different tests, some amplitude values of different cycles have been obtained from the time histories: three first cycle amplitudes for the TPU and twelve first cycle amplitudes for the PEBA. Damping factors were then calculated for all pairs of amplitudes, using Equation (1);

(1) $$\zeta =\frac{\frac{1}{2\pi n}\ln\frac{v_{1}}{v_{2}}}{\sqrt{{\left(\frac{1}{2\pi n}\ln\frac{v_{1}}{v_{2}}\right)}^{2}+1}}$$

where ζ is the damping factor, and v_1_ and v_2_ are two amplitudes separated by n periods [17]. For each specimen, an average has been made with all damping values calculated with all pairs of cycles.

Table 4 shows the averaged values corresponding to each material (average values of the three specimens) with their corresponding deviation. Note that the values are remarkably independent of the free length of the specimen.

### 3.3. Materials Comparison

A comparison between both materials (PEBA90A and TPU98A) is made as the study and the analysis has been the same one for the two materials, thus, the differences between PEBA and TPU are easily pointed out. 

Concerning the printing parameters:

- PEBA deposits less effective material than TPU for the same printing conditions.
- TPU geometrically behaves more like a thermoplastic than PEBA.
- Considering that a honeycomb infill has been used in this work, PEBA resulted in better rigidity and elastic deformation by using a layer height of 0.3 mm. However, TPU shows better results with 0.2 mm layer height. This is a result of different events: as PEBA does not show a big difference between the material deposited and neck formation when using a 0.2 mm or 0.3 mm layer height, and honeycomb presents fibers that work against bending forces, a higher rigidity is found when the original filament has a higher height. If there are no necks created with the 0.3 mm layer height (that it is the case of TPU), the flexural effect shows better results when the layers act as a unique block.
- PEBA results are easier to be printed.

**Figure 9:**
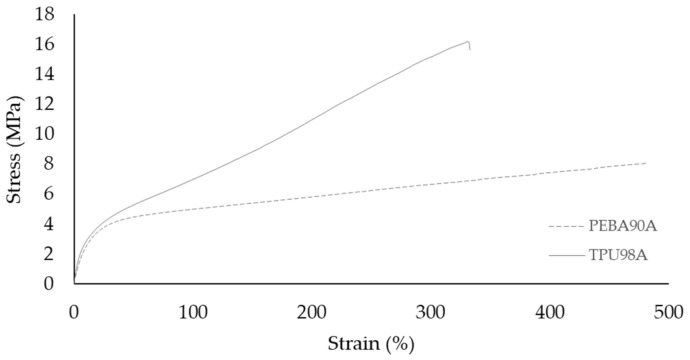
Comparison of TPU 98A and PEBA 90A.

Concerning their mechanical responses:

- All values obtained result in the same magnitude for both materials. Young’s modulus and yield strength for thermoplastic elastomers are expressed in MPa but for thermoplastic materials are in GPa [2,18].
- PEBA can achieve higher deformations but, in general, TPU has a higher rigidity, as it results in higher values of the Young’s modulus. This can be seen in Figure 9. In terms of deformation, PEBA supports higher strains before failure than TPU. However, TPU can achieve higher tensile forces.
- The inter and intra boding of thermoplastic elastomers might be a problem to manufacture the samples, as the optimal creation of necks just appear in certain printing conditions. According to Koo et al. [11], the difficulty might be due to the quick solidification of this type of materials. It can also be due to their retention of elastic behavior after extrusion. Further research should be undertaken to prove this observation in the materials used in this research.

Concerning the differences between the raw materials:

- TPU shows much higher damping capabilities than PEBA, as the vibrating signal becomes indetectable much faster in TPU than in PEBA.
- Both materials have a large range of working temperatures. TPU is characterized for working at high temperatures, up to 90 °C. PEBA can work to low temperatures, capable of working at −60 °C.
- Because of their nature, each material is meant to be used in different fields of applications. Due to its high energy returns, PEBA can be used to manufacture shoe insoles and ski boot fasteners, as they work good in low temperatures [19]. In contrast, TPU can be used to manufacture prosthesis [20].

## 4. Conclusions

The influence of the printing parameters of two different thermoplastic elastomers manufactured by MEX were studied in this paper. Tensile tests were done to obtain the stress-strain curve for each configuration. In addition, the statistical influence of the layer height and infill were studied, resolving an ANOVA. The results observed were explained by checking the actual deposition of materials in forming the layers of the testes specimens, observing that PEBA is less sensitive to layer height change, possibly because of its different viscoelastic properties compared to TPU. 

Results suggest that PEBA and TPU should be treated as different materials, analyzing the effect of printing parameters on the mechanical response shown by them. It is usually assumed that in commercially available thermoplastics, such as PLA, the best mechanical behavior is achieved by setting a low layer height [1,5]. However, this estimation is not always appropriate in thermoplastic elastomers. TPU shows better results of tensile tests when a lower layer height is selected, but not PEBA. 

When both materials are printed in the same conditions (i.e., 100% infill and 0.2 mm layer height), the quantity of material in the effective area for TPU is higher than for PEBA, 95% and 84% of effective material area, respectively, as they show different viscosity. Moreover, when layer height is increased, TPU significantly decreases its effective area whereas this variation is not noteworthy in PEBA.

From the tensile tests, it was observed that the layer height and the fill density are statistically significant parameters for both materials. In TPU’s case, Young modulus and yield strength achieve higher values with 0.2 mm layer height and 75% infill. However, to maximize the mechanical responses for PEBA, the optimal combination is 0.3 mm layer height and 75% infill. Therefore, the proposed methodology can be used for both thermoplastic materials and elastomeric thermoplastics, although the results should be interpreted independently.

In addition, a method for evaluating the damping factor of FFF materials is proposed. The calculated values of damping factors for PEBA and TPU are ζ_PEBA_ = 0.017 and ζ_TPU_ = 0.154, respectively. The damping factor calculated does not depend on the length of the specimens. The vibrating signal absorbed with TPU fades out faster than with PEBA.

## Figures and Tables

**Table 1:** Manufacturing parameters and levels used for the factorial 2-level DoE with a central point.

Parameter/Level	Low	Medium	High
Layer height	0.20	0.25	0.30
Fill density	25	50	75

**Table 2:** Comparison of printing configurations for TPU 98A and PEBA 90A.

Layer Height	TPU 98A	PEBA 98A
0.20 mm	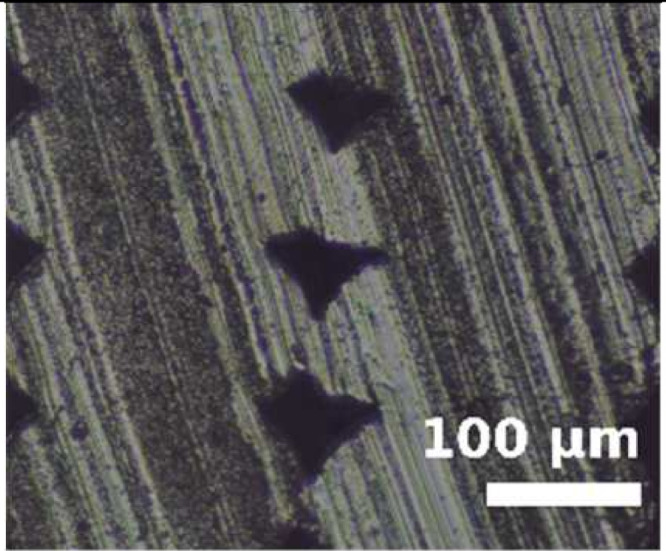	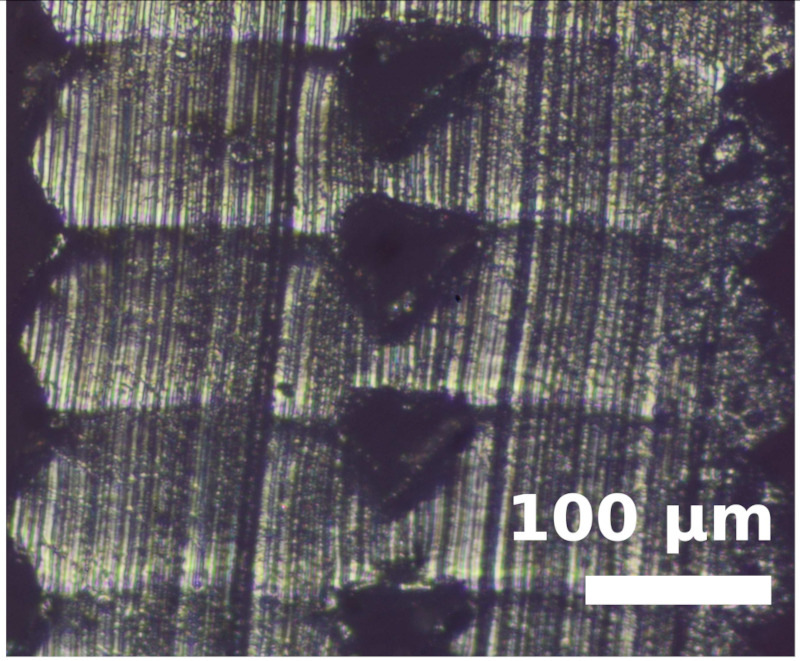
0.25 mm	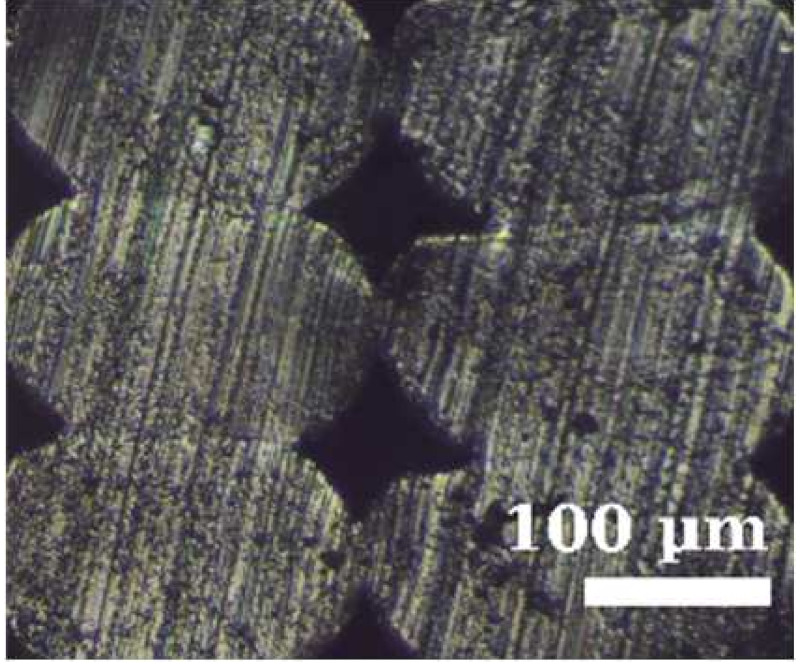	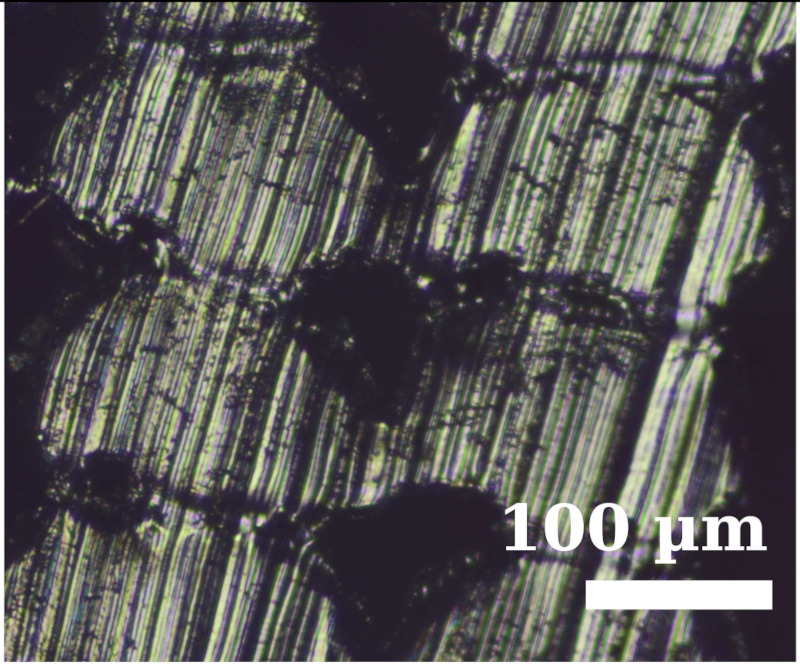
0.30 mm	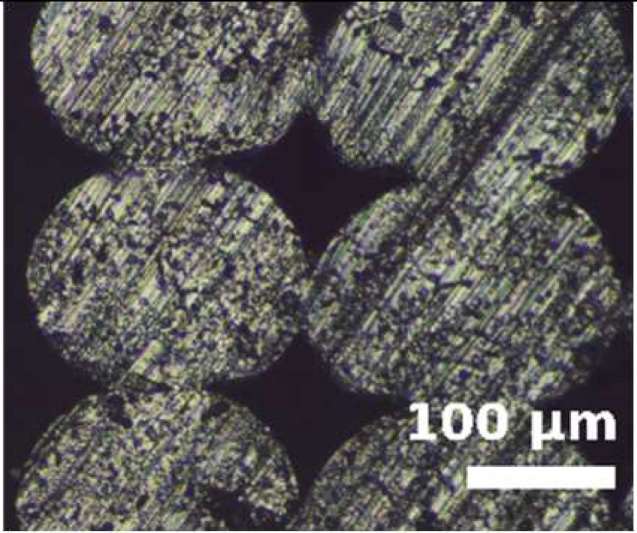	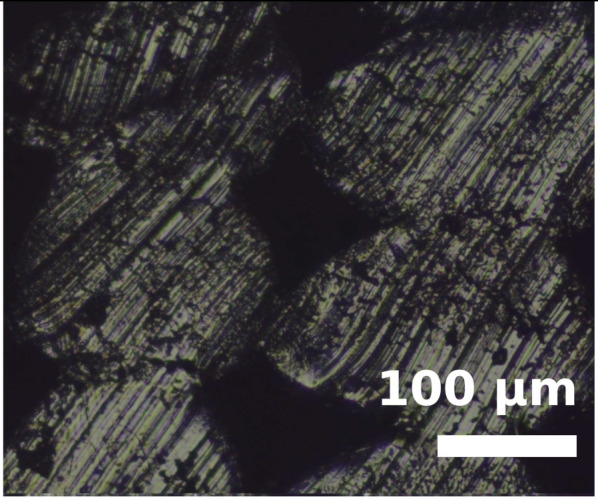

**Table 3:** Average values and standard deviation of tensile tests for TPU 98A and PEBA 90A.

Material	Combination Set		E (MPa)	Rp_0.2_ (MPa)
	Layer Height (mm)	Fill Density (%)		
TPU 98A	**0.2**	25	57.81 ± 2.80	0.67 ± 0.04
	0.2	75	101.10 ± 1.70	0.92 ± 0.08
	0.3	25	68.71 ± 14.69	0.61 ± 0.03
	0.3	75	66.82 ± 15.37	0.73 ± 0.05
	0.25	50	72.81 ± 5.80	0.67 ± 0.04
PEBA 90A	0.2	25	27.63 ± 1.58	0.63 ± 0.03
	0.2	75	32.92 ± 1.48	0.80 ± 0.03
	0.3	25	39.16 ± 1.51	0.85 ± 0.05
	0.3	75	41.75 ± 2.97	0.97 ± 0.04
	0.25	50	34.76 ± 2.41	0.97 ± 0.12

**Table 4:** Averaged damping factors.

Material	Free Length (mm)		
	60	70	Average
PEBA	0.018 ± 0.001	0.017 ± 0.001	0.017
TPU	0.153 ± 0.002	0.156 ± 0.006	0.154

## Data Availability

Not applicable.
